# A23 IMPAIRMENT OF INTESTINAL CRYPTAL STEM CELL CYCLE AND PANETH CELL PROLIFERATION BY CHRONIC STRESS

**DOI:** 10.1093/jcag/gwad061.023

**Published:** 2024-02-14

**Authors:** X Bai, E Ihara, Y Ogawa

**Affiliations:** Medicine, Kyushu Daigaku, Fukuoka, Fukuoka, Japan; Medicine, Kyushu Daigaku, Fukuoka, Fukuoka, Japan; Medicine, Kyushu Daigaku, Fukuoka, Fukuoka, Japan

## Abstract

**Background:**

The small intestine’s epithelial barrier is essential for intestinal homeostasis and host defense. It consists of epithelial cells that are constantly renewed by crypt stem cells and their progeny, which have different functions in the gut. Chronic stress can impair the barrier by modulating the gut-brain axis, which involves the hypothalamic-pituitary-adrenal (HPA) axis, the vagus nerve, and the immune system. Chronic stress can also alter the gut microbiota, which can further influence the barrier and immune system.

**Aims:**

The present study examines how chronic stress affects the crypt stem cell niche and function in the small intestine.

**Methods:**

Restraint stress was applied to 6-8 weeks old mice in a plastic container for 1 hour daily for 7 days. The dark/light transition box test was used to evaluate the stress-induced behavior change. The Ussing chamber technique was used to assess the paracellular permeability of the small intestine. RNA was extracted from the mucosa and submucosa layers of the small intestine, after removing the muscle layer, and RNA sequencing analysis was performed. Immunofluorescence analysis was also performed on the small intestinal tissues to detect the expression of various markers. To assess the *in vivo* effect of cortisol, dexamethasone (a synthetic steroid activating same receptors as cortisol), or metyrapone (an 11β-hydroxylation blocker to inhibit cortisol production), was applied to the mice.

**Results:**

We found that restraint stress induced anxiety-like behavior, increased small intestinal epithelial permeability, and reduced villus and crypt length in mice. RNA sequencing analysis revealed that restraint stress upregulated steroid-driven pathways and downregulated cell cycle and cell mitosis pathways in the small intestinal mucosa, suggesting increased cortisol production and decreased stem cell activity. Immunofluorescence analysis confirmed that restraint stress reduced the expression of stem cell marker Olfm-4 and proliferation markers Ki-67 and p-H3 in the small intestinal crypts. Dexamethasone, a synthetic glucocorticoid, mimicked the effects of restraint stress on crypt stem cell proliferation, while metyrapone, a steroid synthesis inhibitor, attenuated the effects of restraint stress on crypt stem cell proliferation. Restraint stress and dexamethasone also reduced Paneth cell RNA expression and marker in the small intestine.

**Conclusions:**

Chronic stress induces anxiety, barrier dysfunction, and epithelial shortening. Chronic stress also alters crypt stem cell and Paneth cell expression. These findings show how chronic stress impacts intestinal epithelial renewal and differentiation, which may affect intestinal health and disease.

**Funding Agencies:**

JSPS

